# An ensemble machine learning model assists in the diagnosis of gastric ectopic pancreas and gastric stromal tumors

**DOI:** 10.1186/s13244-024-01809-2

**Published:** 2024-09-19

**Authors:** Kui Sun, Ying Wang, Rongchao Shi, Siyu Wu, Ximing Wang

**Affiliations:** 1 Department of General Surgery, Peking University Third Hospital, 49 North Garden Road, Haidian District, Beijing, 100191 China; 2 Department of Radiology, Shandong Provincial Hospital Affiliated to Shandong First Medical University, Jing Wu Road, No. 324, Jinan, 250021 China; 3 School of Radiology, Shandong First Medical University & Shandong Academy of Medical Sciences, Taian, 271016 China; 4 Department of Radiology, Beijing Friendship Hospital, Capital Medical University, Beijing, China; 5 Department of Radiology, Shandong Provincial Hospital, Shandong University, Jing Wu Road, No. 324, Jinan, 250021 China

**Keywords:** Gastric ectopic pancreas, Gastric stromal tumors, Deep learning, Radiomics, Multiphase computed tomography

## Abstract

**Objective:**

To develop an ensemble machine learning (eML) model using multiphase computed tomography (MPCT) for distinguishing between gastric ectopic pancreas (GEP) and gastric stromal tumors (GIST) in lesions < 3 cm.

**Methods:**

In this study, we retrospectively collected MPCT images from 138 patients between April 2017 and June 2023 across two centers. Cohort 1 comprised 94 patients divided into a training cohort and an internal validation cohort, while the 44 patients from Cohort 2 constituted the external validation cohort. Deep learning (DL) models were constructed based on the lesion region, and radiomics features were extracted to develop radiomics models, which were later integrated into the fusion model. Model performance was assessed through the analysis of the area under the receiver operating characteristic curve (AUROC). The diagnostic efficacy of the optimal model was compared with that of a radiologist. Additionally, the radiologist with the assistance of the eML model provides a secondary diagnosis, to assess the potential clinical value of the model.

**Results:**

After evaluation using an external validation cohort, the radiomics model demonstrated the highest performance in the venous phase, achieving AUROC of 0.87. The DL model showed optimal performance in the non-contrast phase, with AUROC of 0.81. The eML achieved the best performance across all models, with AUROC of 0.90. The use of eML-assisted analysis resulted in a significant improvement in the junior radiologist’s accuracy, rising from 0.77 to 0.93 (*p* < 0.05). However, the senior radiologist’s accuracy, while improving from 0.86 to 0.95, did not exhibit a statistically significant difference.

**Conclusion:**

eML model based on MPCT can effectively distinguish between GEPs and GISTs < 3 cm.

**Critical relevance statement:**

The multiphase CT-based fusion model, incorporating radiomics and DL technology, proves effective in distinguishing between GEP and gastric stromal tumors, serving as a valuable tool to enhance diagnoses and offering references for clinical decision-making.

**Key Points:**

- No studies yet differentiated these tumors via radiomics or DL.
- Radiomics and DL methodologies unveil potentially distinct phenotypes within lesions.
- Quantitative analysis on CT for GIST and ectopic pancreas.
- Ensemble learning aids accurate diagnoses, assisting treatment decisions.

**Graphical Abstract:**

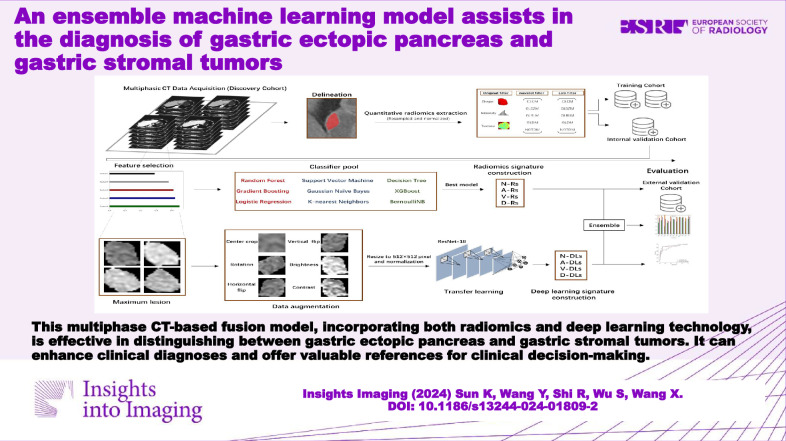

## Introduction

Gastric ectopic pancreas (GEP) is a congenital anomaly characterized by anomalous pancreatic tissue that is anatomically and vascularly distinct from the primary pancreatic body [1]. Analogous to sediment deposits during embryonic development in the gastrointestinal tract [2], most GEP cases are asymptomatic and are typically discovered incidentally during unrelated abdominal surgeries or post-mortem examinations, presenting a challenge for accurate prevalence estimation. The likelihood of intraoperative discovery is approximately 0.2% [3, 4]. GEP can manifest anywhere along the gastrointestinal tract, and it may also be found in the gallbladder, spleen, mediastinum, and other tissues. According to surgical and autopsy reports, the stomach is the predominant site for GEP, accounting for approximately 52% of cases [5].

Diagnosing GEP presents challenges owing to its rarity, variability, and non-specific clinical presentation. Studies report that over 54% of GEP cases are misdiagnosed before surgery [4]. When GEP occurs in the stomach, it typically manifests as a submucosal mass, a characteristic that can create confusion with gastric stromal tumors (GIST) during gastroscopy or imaging examinations. GISTs, the most prevalent type of gastric submucosal tumors, exhibit aggressiveness and carry a 50% risk of metastasis [6]. The primary treatment for GISTs involves surgical resection. In contrast, GEP constitutes a benign subepithelial lesion. Furthermore, distinguishing GEP from GIST is clinically significant, as opting for follow-up without surgical excision may be suitable for GEP cases, unlike the need for surgical intervention in GIST cases [7, 8].

Computed tomography (CT) has emerged as the most used non-invasive modality for the preoperative evaluation of gastric tumors, owing to its rapid imaging capabilities and widespread availability [9]. The presence of pancreatic ductal and acinar glandular components in GEP, causes their contrast enhancement pattern is very similar to that of the pancreas and is a crucial criterion for CT diagnosis [10]. However, in practical clinical settings, GEP lesions are often small, and these CT features can prove challenging to discern. With the increasing prevalence of CT usage, an escalating number of GEP cases are being scanned, amplifying the challenge in clinical practice due to the similar CT manifestations of GEP and GIST. Particularly, GISTs with a diameter < 3 cm manifest as solid masses of uniform density [11].

Radiomics extracts numerous quantitative features from medical images, enabling the quantitative assessment of tumor heterogeneity. In recent years, it has demonstrated outstanding performance in diagnosing, staging, and predicting outcomes for various malignancies [12, 13]. Deep learning (DL), an emerging branch of machine learning (ML), captures phenotypic differences in diseases that may not be captured by radiomics. The heterogeneity existing and reflected in lesions in images is explored by means of a neural network, thereby enhancing disease diagnosis [14, 15].

Therefore, the study aims to create and validate a CT-based fusion model that integrates radiomics and DL techniques to effectively differentiate between GEP and GIST and assist in improving clinical diagnostic efficacy.

## Method and materials

### Patients

In this retrospective two-center study, we developed an ensemble ML (eML) analysis to distinguish between GEP and GIST. The model underwent training on a discovery cohort and subsequent validation using an external cohort. CT data from patients diagnosed with GEP and GIST, confirmed by pathological examination, were retrieved from April 2017 to June 2023. Baseline patient information, encompassing age, gender, lesion location, and resection approach, was extracted from medical records. The study received approval from the Institutional Review Board, and the requirement to obtain informed consent was waived.

**Fig. 1:**
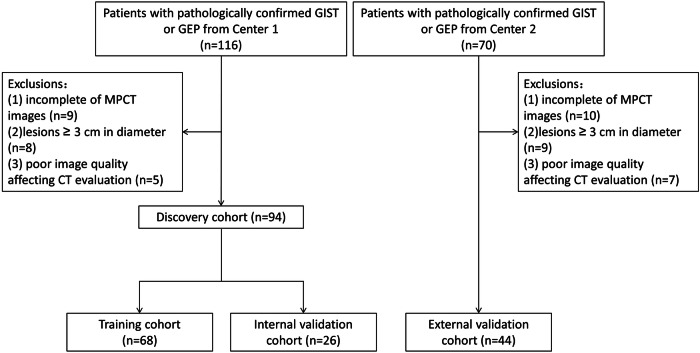
Study flowchart in the multicenter cohorts. GIST, gastric stromal tumors; GEP, gastric ectopic pancreas; MPCT, multiphase CT

Patients who fulfilled the following criteria were included: (1) comprised lesions detectable on CT images (diameter > 1 cm) primarily composed of solid components [11], and (2) lesions pathologically confirmed as GIST or GEP. The exclusion criteria were as follows: (1) incomplete MPCT images (non-contrast, arterial, venous, and delayed phases); (2) lesions ≥ 3 cm in diameter; and (3) poor image quality affecting CT evaluation. The inclusion and exclusion workflow are illustrated in Fig. 1.

### MPCT image data acquisition protocol

The CT examinations were conducted using either the Siemens Force dual-source 128-row CT scanner or the Siemens Definition Flash dual-source 128-row CT scanner, employing the following parameters: a tube current of 100 mAs, tube voltage of 120 kV, a matrix size of 512 × 512, pitch of 1.5, and layer thickness of 1.5 mm. Contrast agent enhancement was achieved using the intravenous group injection tracking method, with iodophoresis injection (Shanghai Stellite) having an iodine concentration of 350 mg/mL, a dose of 80 mL, and a flow rate of 2.3 mL/s. Scans were delayed for 30, 60, and 120 s for the arterial phase, venous phase, and delayed phase, respectively, after injection. For stomach scans, all patients were advised to fast overnight and drink 600–1000 mL of warm water to distend the stomach before the CT examination.

### Manual delineation manner

MPCT images were retrieved and collected from the Picture Archiving and Communication System (PACS). Unlike parenchymal organs, the stomach is influenced by gastric peristalsis and gas, leading to challenges in CT image registration performance. Consequently, two junior radiologists (Y.W. and Rc.S. with five years of experience in abdominal radiology diagnosis) independently delineated regions of interest (ROI) manually in respective single CT phase, by using ITK-Snap software version 4.0.2 (http://www.itksnap.org). The results of ROI delineation were subsequently double-checked by senior radiologists with 20 years of experience in abdominal radiology. In instances of disagreement, resolution or re-delineation of the ROI was determined through negotiation.

### MPCT image preprocessing and radiomics feature extraction

MPCT images were preprocessed using the SimpleITK toolbox version 2.1.1 (https://simpleitk.org). The images were resampled to 2 × 2 × 2 mm³ voxels using the B-spline method, and the CT Hounsfield units (HUs) were discretized into 70 bins ranging from −1000 to 3000 HUs. The extracted features encompassed three-dimensional geometric shapes, gray intensity, and textural features. Textural features included the gray level co-occurrence matrix, gray level size zone matrix, gray level run length matrix, gray level dependence matrix, and neighboring gray tone difference matrix. In addition, it included textural features processed by filters such as Wavelet and Laplacian of Gaussian (LoG). Twenty patients were randomly selected and conducted re-segmentation. Radiomics features consistency test was performed by using an interclass correlation coefficient (ICC), the ICC > 0.75 was considered as high consistency. In addition, ICC was used to evaluate the diagnostic consistency between radiologists of different experience levels with and without the aid of a predictive model.

### Radiomics feature selection and ML construction

The discovery cohort was randomly divided into training (*n* = 68) and internal validation (*n* = 26) cohorts before selection. Feature contribution in the random forest (RF) algorithm was utilized as the screening metric for feature selection. To address the small sample scale and potential overfitting caused by a large number of features, we limited the optimal feature quantity range to 1–20, i.e., no more than 1/4 of the total sample size. The best number of features was determined through the evaluation of the internal validation cohort.

Given the diversity of ML algorithms and the varying prediction performances they yield when fitted to the same dataset, multiple classical ML classifiers were employed in this study. The classifiers included RF, gradient boosting, logistic regression, support vector machine with linear and radial basis function kernels, Gaussian naïve Bayes, K-nearest neighbors, decision tree, XGBoost, and BernoulliNB. Model performance was assessed using the area under the receiver operating characteristic curve (AUROC). On the external validation cohort, the highest AUROC achieved by any of the multiple ML models was considered the best model with good generalization to build the radiomics signature (Rs).

### Deep learning model construction

The ResNet-18, pre-trained on the natural image library ImageNet (http://www.imagenet.org), served as the backbone DL neural network in this study, employing a transfer learning strategy. The weights pre-trained by ImageNet were loaded into the DL model, and gradient and weight information were updated by fitting the training data of this study to achieve the medical classification objective.

Prior to analysis, the maximum lesion of the two diseases in MPCT images was extracted using a computational method. To address the sample size issue, data augmentation was applied to the training cohort. This strategy allows the model to learn semantic information expressed by different images and prevents overfitting. Six augmentation methods were used, including center crop, random rotation, random horizontal flip, random vertical flip, and random color changes in brightness and contrast. Subsequently, the images were resized to 512 × 512 pixels to standardize the distance scale, and pixel values were normalized to *Z*-score for further analysis.

The training cohort served as input to fit the DL model, and the internal validation cohort was used for hyperparameter tuning. Once optimal hyperparameters were determined for the DL model, the external validation cohort was used to evaluate generalization and generate the DL signature (DLs). The chosen hyperparameters included the Adaptive Moment Estimation optimizer with betas ranging from 0.9 to 0.999, epsilon set to 1e-8, batch sizes of 16, 100 epochs, and a learning rate of 3e-4. The loss function used to train the network was CrossEntropyLoss.

### eML model construction

**Fig. 2:**
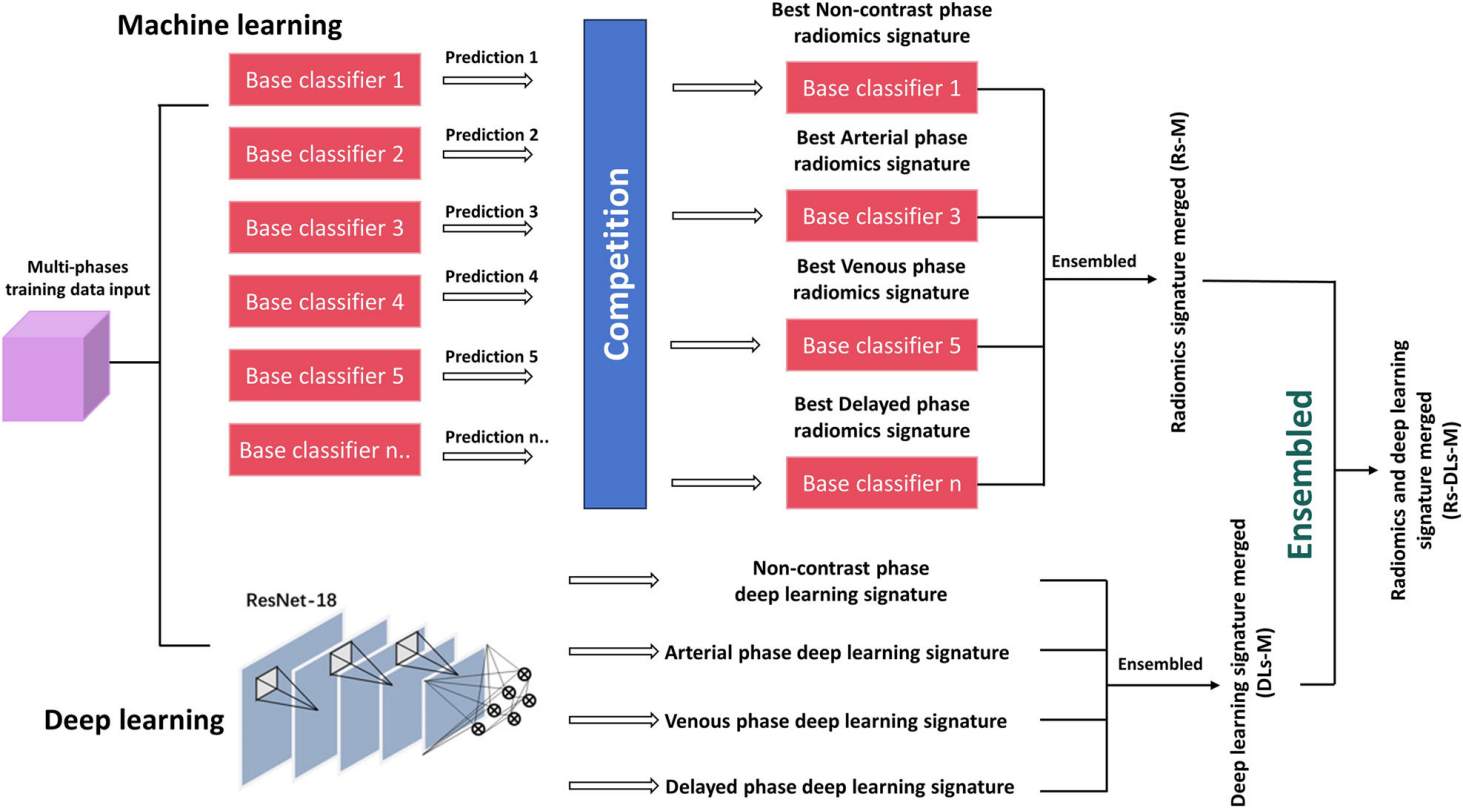
Ensemble machine learning (eML) workflow

The Radiomics Signature Merged (Rs-M) model was constructed by combining Rs generated by the best ML model for each phase. Similarly, the DL Signature Merged (DLs-M) model was created by combining DLs generated by the well-trained DL model for each phase. The final Radiomics and Deep Learning Signature Merged (Rs-DLs-M) model comprised Rs-M and DLs-M. In total, eleven models were built, including four radiomics models for each phase (Non-contrast Rs, N-Rs; Artery Rs, A-Rs; Venous Rs, V-Rs; Delayed Rs, D-Rs), four DL models for each phase (Non-contrast DLs, N-DLs; Artery DLs, A-DLs; Venous DLs, V-DLs; Delayed DLs, D-DLs), Rs-M, DLs-M, and Rs-DLs-M. The eML workflow is illustrated in Fig. 2.

### Radiologist reading and model assist test

A two-stage radiologist study was conducted to evaluate the diagnostic performance of Rs-DLs-M and assess its clinical application value. Two anonymous radiologists, one with seven years and the other with twenty years of experience in abdominal imaging, participated in this study. The external validation cohort was shuffled and presented to the radiologists. Each radiologist was asked to interpret the images blindly and independently.

In the first stage of the radiologist study, both the MPCT images and baseline characteristics of each patient were provided to the radiologists for diagnosis. In the second stage (eML-assisted radiologist), corresponding probabilities were supplied to the radiologists. In this stage, each radiologist had the option to either change or maintain the initial diagnosis, providing their final diagnostic conclusions. The radiologists remained blind to the pathological results until the completion of the two-stage radiologist study.

### Statistical analysis

Statistical analysis was performed in the R software version 4.1.2 (https://www.r-project.org). For the continuous variables, Shapiro-Wilk test was applied to distribution test. If the variables conformed to a normal distribution, the Student *t*-test was applied to the analysis, otherwise, the Wilcoxon test was applied. For the categorical variables, Chi-square or Fisher exact tests (if applicable) are used, *p* < 0.05 was considered a significant statistical difference. The AUROC and its 95% confidence interval, accuracy, sensitivity, specificity, and F1-socre were used to evaluate the efficacy of models. The Delong test was employed to evaluate statistical differences in diagnostic efficiency, with *p* < 0.05 indicating statistical significance. The maximum lesion was extracted by using SimpleITK version 2.1.1 (https://simpleitk.org) and Opencv version 4.5.1 (https://opencv.org). The DL framework was constructed using PyTorch version 1.9.0 (https://pytorch.org). The computer core hardware of the study used a CPU with Intel Core I9-10900K, a GPU with Nvidia RTX 3090 24GB, and a RAM with Kingston DDR4-3200 32GB.

## Results

### Patient baseline

**Fig. 3:**
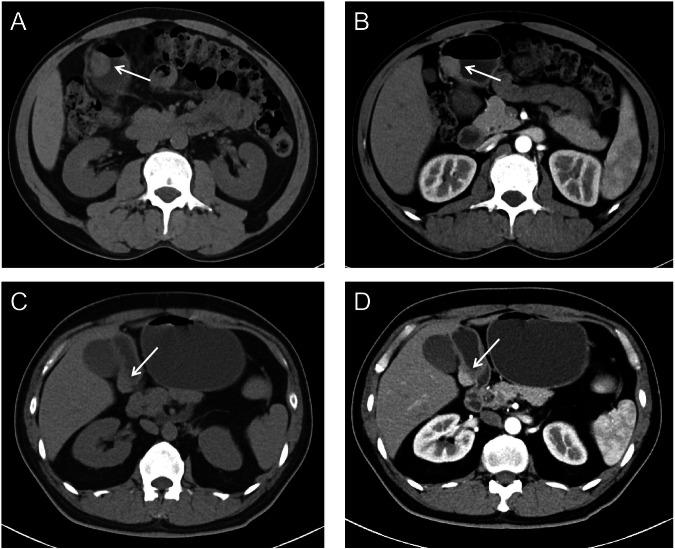
Representative multiphase CT images of gastric ectopic pancreas (GEP) and gastric stromal tumors (GIST, white arrows). **A** non-contrast phase of GEP, **B** arterial phase of GEP, **C** non-contrast phase of GIST, **D** arterial phase of GIST

A total of 138 patients from two centers were enrolled in this study, with 94 from Center 1 forming the discovery cohort and 44 from Center 2 serving as the external validation cohort. The discovery cohort comprised 94 patients (training and internal validation cohorts) from Center 1, while the external validation cohort comprised 44 patients from Center 2. In the training cohort, there were 40 patients with GIST and 28 patients with GEP, while the internal validation cohort included 14 GISTs and 12 GEPs. The median age of the patients in the discovery cohort was 57 years (range, 20–77 years). For the external validation cohort, there were 29 GISTs and 15 GEPs, with a median age of 59 years (range, 40–78 years). Demographics of patients are shown in Table 1 and the illustration of GEP and GIST is shown in Fig. 3.

**Table 1:** The baseline characteristics of patients

Baseline characteristics	Center 1 (*n* = 94)	Center 2 (*n* = 44)
Age (years)	57 (48.3, 62.0)	59 (53.5, 67.0)
Male	45 (47.9%)	25 (56.8%)
Resection approach		
Gastrectomy under laparoscopic surgery	15 (16.0%)	6 (13.6%)
Conventional gastrectomy	7 (7.4%)	3 (6.8%)
Gastrointestinal endoscopic surgery	72 (76.6%)	35 (79.6%)
Stomach location		
Upper	30 (31.9%)	18 (40.9%)
Middle	41 (43.6%)	19 (43.2%)
Lower	23 (24.5%)	7 (15.9%)

### Optimal features component

**Fig. 4:**
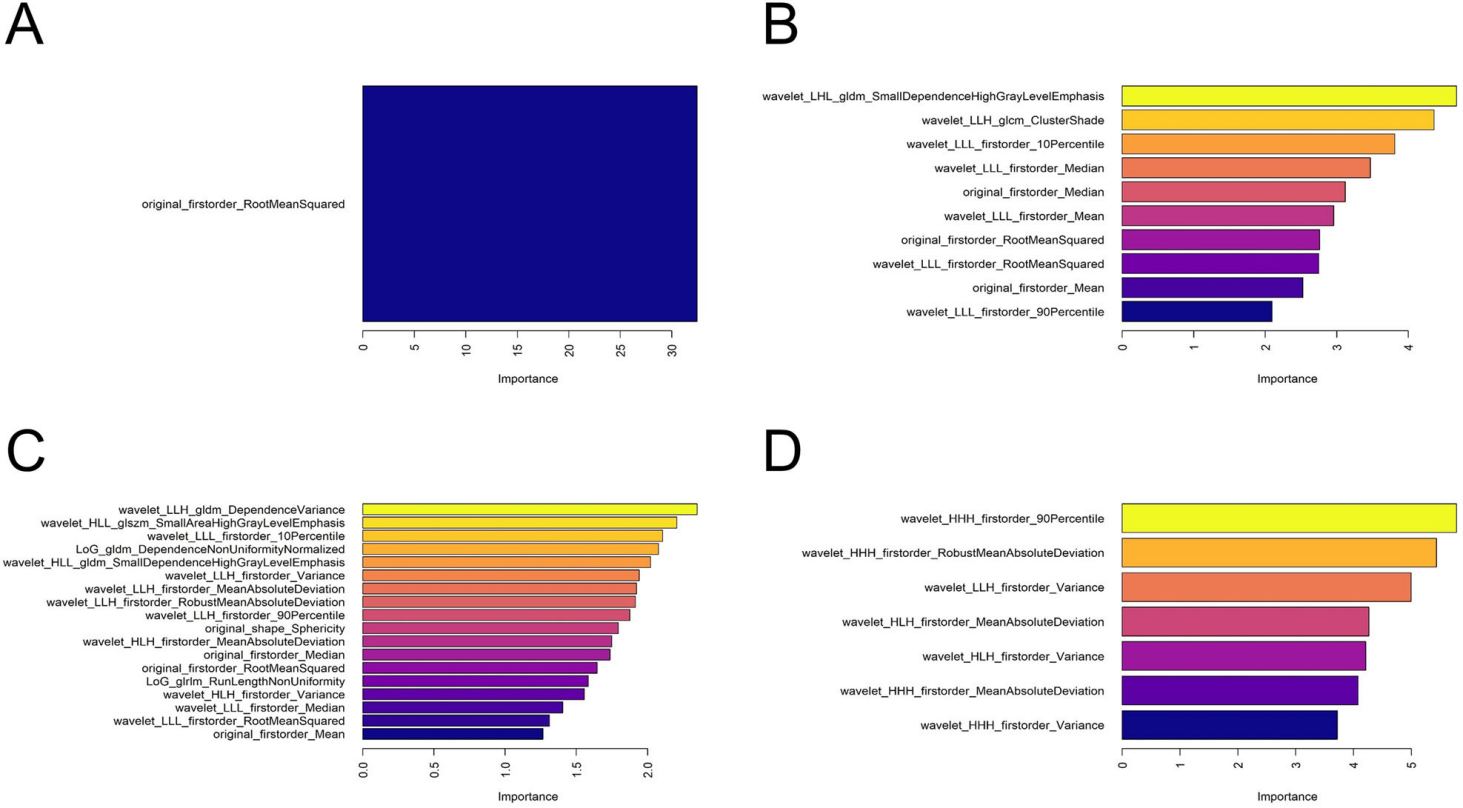
The optimal features distribution and its contribution (importance) on (**A**) non-contrast phase, (**B**) arterial phase, (**C**) venous phase, and (**D**) delayed phase of radiomics model

In the N-Rs, only a singular intensity feature processed by the original filter persisted. Within the A-Rs, ten features, consisting of eight intensity and two texture features, were preserved. Of these, seven underwent processing via a wavelet filter, while the remaining three were processed using the original filter. In the V-Rs, 18 features, encompassing 12 intensity, 5 texture, and one shape feature, were identified as optimal. Among these, 12 features were subjected to wavelet filter processing, 2 to LoG filter, and 4 to the original filter. Concerning the D-Rs, it is exclusively comprised of seven intensity features, all of which undergo processing using a wavelet filter. The optimal features distribution and its contribution (importance) are shown in Fig. 4. The result of the ICC test for optimal features is shown in Table S1.

### Rs performance

In the radiomics analysis, each phase of the CT image exhibits potential predictive value. The results indicated that the venous phase demonstrated the highest performance, followed by the arterial phase, the non-contrast phase, and finally, the delayed phase. The AUROC values for these phases were 0.87 (0.74–1.00), 0.82 (0.67–0.98), 0.71 (0.51–0.91), and 0.69 (0.53–0.85), respectively. Correspondingly, the accuracies for these phases were 0.91, 0.89, 0.82, and 0.68, respectively. Detailed information is presented in Table 2.

**Table 2:** Comparison of performance between different diagnostic models

Model	AUROC (95% CI)	Acc	Sen	Spe	F1-score
Radiomics					
N-Rs	0.71 (0.51–0.91)	0.82	0.67	0.90	0.71
A-Rs	0.82 (0.67–0.98)	0.89	0.73	0.97	0.81
V-Rs	0.87 (0.74–1.00)	0.91	0.80	0.97	0.86
D-Rs	0.69 (0.53–0.85)	0.68	0.60	0.72	0.56
Deep learning					
N-DLs	0.81 (0.67–0.96)	0.77	0.80	0.76	0.71
A-DLs	0.74 (0.56–0.92)	0.84	0.60	0.97	0.72
V-DLs	0.78 (0.62–0.95)	0.80	0.67	0.86	0.69
D-DLs	0.69 (0.51–0.87)	0.80	0.40	1.00	0.57
Merged					
Rs-M	0.88 (0.75–1.00)	0.88	0.86	0.90	0.84
DLs-M	0.84 (0.71–0.96)	0.84	0.67	0.93	0.74
Rs-DLs-M	0.90 (0.79–1.00)	0.89	0.87	0.90	0.84

*AUROC* area under the receiver operating characteristic curve, *Acc* accuracy, *Sen* sensitivity, *Spe* specificity

### DLs performance

In the DL analysis, the non-contrast phase exhibited superior generalization and robustness, achieving an AUROC of 0.81 (0.67–0.69) and an accuracy of 0.77. Following closely, the venous phase secured the second position, achieving an AUROC of 0.78 (0.62–0.95) and an accuracy of 0.80. The arterial phase occupied the third rank, demonstrating an AUROC of 0.74 (0.56–0.92) and an accuracy of 0.84. Intriguingly, the delayed phase displayed the lowest performance in both radiomics and DL analyses, recording an AUROC of 0.69 (0.51–0.87) and an accuracy of 0.80.

### Merged model performance

**Fig. 5:**
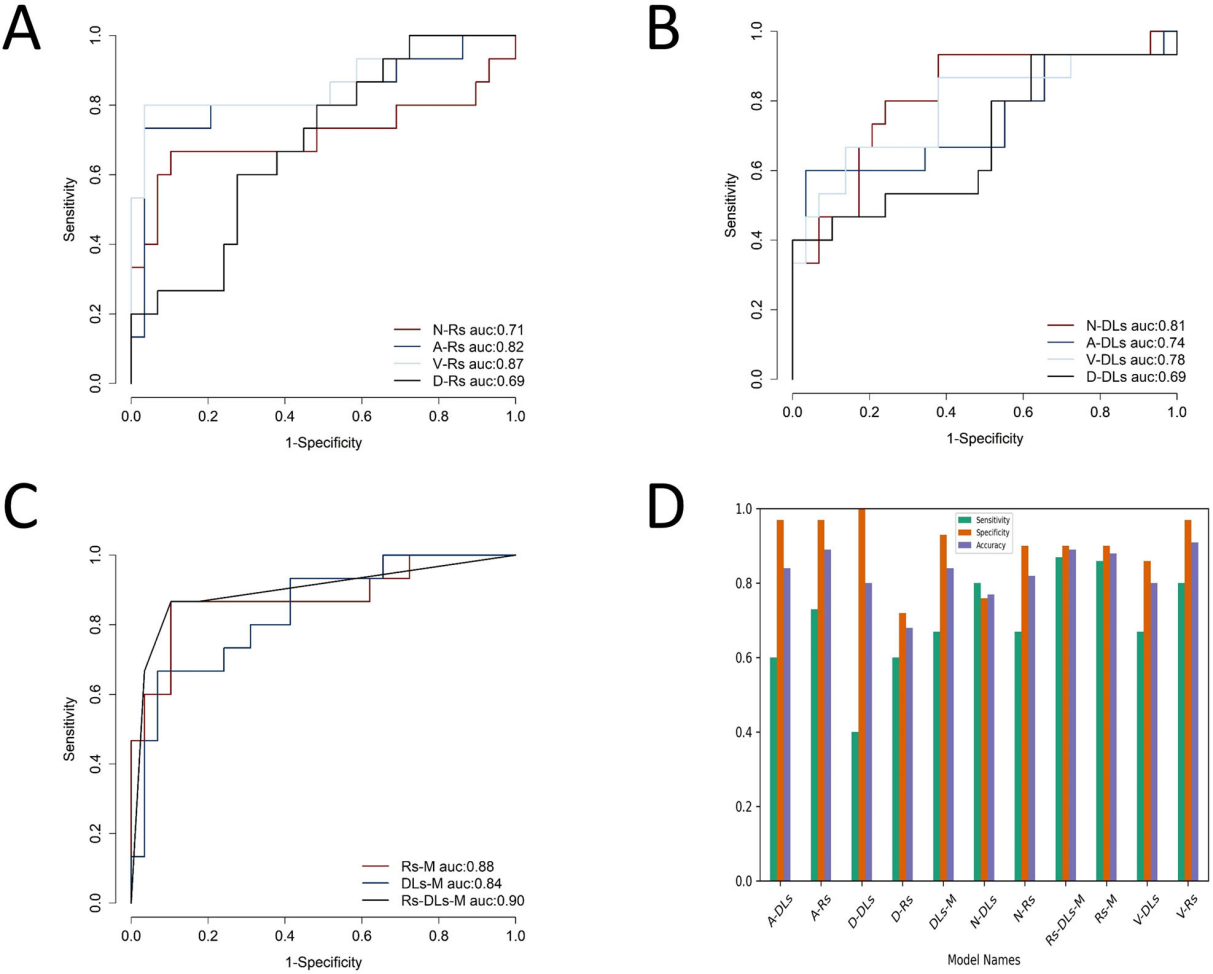
Comparison of the diagnostic efficacy of different models. **A** Comparison of radiomics signature area under the receiver operating characteristic curve (AUROC) of different CT phases, **B** Comparison of deep learning signature AUROC of different CT phases, **C** Comparison of radiomics fusion model, deep learning fusion model, and integrated model AUROC, **D** Columnar graphs of the sensitivity, specificity, and accuracy of different models. N-Rs, noncontrast radiomics signature; A-Rs, artery radiomics signature; V-Rs, venous radiomics signature; D-Rs, delayed radiomics signature; N-DLs, non-contrast deep learning signature; A-DLs, artery deep learning signature; V-DLs, venous deep learning signature; D-DLs, delayed deep learning signature; Rs-M; radiomics signature merged; DLs-M; deep learning signature merged; Rs-DLs-M; radiomics and deep learning signature merged

Three combination methods were employed, namely Rs-M, which amalgamated radiomics features from four phases, DLs-M, combining DL scores from four phases, and Rs-DLs-M, a fusion of Rs-M and DLs-M. These models collectively exhibited commendable comprehensive predictive performance, Rs-DLs-M appears to be more balanced in all aspects of prediction indicators (Fig. 5D), reaching an AUROC of 0.90 (0.79–1.00) and an accuracy of 0.89. Comparative metrics, including accuracy, sensitivity, and specificity, are illustrated in Fig. 5. We used the Delong test to assess whether there was a statistical difference in diagnostic performance between Rs-DLs-M and the two methods’ single-modal optimal models (i.e., V-Rs, N-DLs). The results showed no statistical difference, in which the *p*-value of Rs-DLs-M compared with V-Rs was 0.56, and 0.37 for N-DLs.

In the radiologist diagnostic trial, the initial stage saw an accuracy of 0.77 for junior radiologist and 0.86 for senior radiologist. In the subsequent stage, with the inclusion of eML assistance, diagnostic accuracy improved to 0.93 for junior radiologist and 0.95 for senior radiologist. The results of the Delong test indicated a significant improvement in the comprehensive diagnostic performance of junior radiologists with the assistance of eML (*p* < 0.05). Although senior radiologists also showed improvement, the *p*-value was not statistically significant (Table 3).

**Table 3:** Diagnostic performance of radiologists before and after ensemble machine learning (eML) assistance

	Before			After		
Radiologists/Model	Acc	Sen	Spe	Acc	Sen	Spe
Junior (7-years)^a^	0.77	0.60	0.86	0.93↑	0.87↑	0.97↑
Senior (20-years)^b^	0.86	0.73	0.93	0.95↑	0.93↑	0.97↑
Rs-DLs-M	0.89	0.87	0.90	-	-	-

*Acc* accuracy, *Sen* sensitivity, *Spe* specificity

^a^ Delong-test was used to compare the diagnostic efficiency before and after eML assistance, indicating *p* < 0.05

^b^ Delong-test was used to compare the diagnostic efficiency before and after eML assistance, indicating no statistical difference

The ICC consistency analysis further demonstrated enhancements with eML-assisted diagnosis. Using pathological results as the ground truth, the ICC value for junior radiologists increased from 0.483 to 0.849 with eML assistance. For senior radiologists, the ICC value rose from 0.691 to 0.901. Additionally, the concordance between junior and senior radiologists’ diagnoses improved from 0.747 to 0.786 with eML assistance.

## Discussion

Contrast-enhanced CT is widely regarded as the primary imaging method for detecting abdominal masses [16]. However, the accurate detection of GEP is heavily dependent on the clinical experience of the radiologist, leading to considerable variation in GEP detection rates among radiologists with varying years of experience. A precise diagnosis of GEP is crucial for guiding appropriate treatment strategies. Regrettably, patients often encounter misdiagnoses with GIST before surgery, resulting in unnecessary extensive resections. In this study, we developed eML model incorporating radiomics and DL based on MPCT images. We compared the model’s performance with diagnoses made by radiologists of varying experience levels. Our results demonstrated that both the radiomics and DL models across all phases exhibited discrimination between GEP and GIST. Particularly noteworthy was the fusion model, Rs-DLs-M, which achieved the highest diagnostic performance with an AUROC of 0.90 (0.79–1.00) and an accuracy of 0.89. Moreover, the comprehensive diagnostic capabilities of junior radiologists improved from an initial accuracy of 0.77, sensitivity of 0.60, and specificity of 0.86 to 0.93, 0.87, and 0.97, respectively. Meanwhile, senior radiologist improved from 0.86, 0.73, and 0.93 to 0.95, 0.93, and 0.96, respectively.

GEP masses comprise pancreatic acini, ductal components, and islets in varying proportions. The MPCT features of GEP are intricately linked to the composition of its pathological tissue. The enhancement degree of GEP, where duct structure and muscle layer are the primary components, is often lower than that of the normal pancreas. In cases where GEP is predominantly composed of acinar tissue, the enhancement pattern typically mirrors that of the normal pancreas, presenting a tendency towards uniformity [17, 18]. In contrast, GISTs are true neoplasms constituted by spindle cells, epithelioid cells, or a combination of both. These tumors usually exhibit homogeneity on MPCT images. On CT images, GISTs frequently manifest as exophytic growths, commonly appearing as masses protruding from the gastrointestinal tract wall into the abdominal cavity [19]. Similarly, the characteristic of outward growth can also be observed in GEP. Mucosal ulceration is a common occurrence on the luminal surface of most GISTs, a presentation that may be mistaken for GEP with duct-like structures [20, 21].

The radiomics results highlight the intensity feature as the most influential, with contributions from the non-contrast phase (1/1), arterial phase (8/10), venous phase (12/18), and delayed phase (7/7). This observation led us to hypothesize that the heterogeneity of gray-scale histogram parameters derived from MPCT might be associated with variations in intra-tissue pathological components in GEP and GIST [22]. Coincidentally, Li et al [11] study showed that both lesions exhibited uniform enhancement overall, but the degree of enhancement was higher in the venous phase compared to the arterial phase. Additionally, in quantitative analysis, they found that the enhancement value and enhancement rate of GEP were higher than those of GIST (GEP = 43.54 HU ± 11.78, GIST = 29.16 HU ± 13.69, *p* < 0.001), enhancement ratio (GEP = 1.08 ± 0.45, GIST = 0.77 ± 0.37, *p* < 0.001), with statistical differences observed. This phenomenon suggests that the venous stage plays a more significant role than other stages, highlighting the diagnostic value of intensity features, similar to our hypothesis. It also explains why V-Rs in this study contained more pronounced intensity features and demonstrated the best single-phase prediction performance.

The introduction of eML-assisted diagnosis resulted in a significant improvement in the ICC for junior radiologists from 0.483 to 0.849, and their diagnostic performance increased by 16%, 27%, and 11%, respectively, as evidenced by the Delong test. However, no significant difference was observed for senior radiologists. Junior radiologists, lacking sufficient clinical practice and subject knowledge, require time to develop their skills. The rare conditions addressed in this study demonstrate how eML can help junior radiologists diagnose these cases more quickly, shortening their learning curve and enhancing their clinical experience. In contrast, senior radiologists, having gained extensive experience through long-term clinical practice, have a greater ability to recognize various diseases. This experience plays an important role in the result, which means that eML assistance, while helpful, is less impactful for them.

In contrast to other studies focusing on the subjective analysis of MPCT for differentiating GIST and GEP, our investigation relies on an objective assessment through quantitative radiomics analysis of the entire lesion ROI for differential diagnosis. This analytical approach spans the entirety of the tumor, providing more comprehensive three-dimensional information that reflects the heterogeneity of GIST and GEP. This stands in contrast to the limitations posed by single-slice CT images, which offer only two-dimensional information. It is worth noting that this may potentially explain the slightly inferior performance of the DL models in our study. Kim et al [9] described the best predictor of MPCT-based signs for diagnosing GEP, with a sensitivity of 0.857 and specificity of 0.825. However, this performance is surpassed by our fusion model, which achieved a sensitivity of 0.87 and specificity of 0.90. In a single-center study by Li et al [11], focusing on the identification of GEP and GIST using CT images based on 132 individuals, the enhancement ratio to the pancreas was identified as the best predictor, with an AUROC of 0.81 (0.71–0.88). Our fusion model validated by external cohort, in contrast, demonstrated superior performance with an AUROC of 0.90 (0.79–1.00).

This study has several limitations. First, being a retrospective study, there is a possibility of selection bias. Second, despite the inclusion of samples from two centers, the overall sample size remains relatively small. Larger prospective investigations may be necessary to validate the current findings in the future. Third, manual segmentation is time-consuming, and a fully automated segmentation method is urgently needed to increase efficiency.

## Conclusion

We developed and validated an eML model based on non-invasive MPCT to automatically identify GEP and GIST. This method holds the potential to assist radiologists in achieving prompt and accurate diagnoses, serving as a valuable reference for decision-making.

## Supplementary information

ELECTRONIC SUPPLEMENTARY MATERIAL

## Data Availability

The datasets used and/or analysed during the current study are available from the corresponding author on reasonable request.

## Abbreviations

AUROC: Area under the receiver operating characteristic curve
DL: Deep learning
eML: Ensemble machine learning
GEP: Gastric ectopic pancreas
GIST: Gastric stromal tumors
LoG: Laplacian of Gaussian
MPCT: Multiphases computed tomography
RF: Random forest
ROI: Regions of interest
Rs: Radiomics signature
