# Supplementary material for: An ensemble machine learning model assists in the diagnosis of gastric ectopic pancreas and gastric stromal tumors
Source: Insights Imaging. 2024 Sep 19;15:225. doi: 10.1186/s13244-024-01809-2 (PMC11424595; doi:10.1186/s13244-024-01809-2)
Supplement: Supplementary file 1 — ELECTRONIC SUPPLEMENTARY MATERIAL [file 13244_2024_1809_MOESM1_ESM.pdf]

An ensemble machine learning model assists in the diagnosis of gastric ectopic pancreas and gastric stromal tumors

ELECTRONIC SUPPLEMENTARY MATERIAL

Table S1. The interclass correlation coefficient test for best radiomics features on each phase model

Model	Filter	Class	Name	ICC
N-Rs	Original	Firstorder	RootMeanSquared	0.856
A-Rs	Wavelet-LLL	Firstorder	Median	0.986
	Wavelet-LLL	Firstorder	Mean	0.967
	Original	Firstorder	RootMeanSquared	0.980
	Wavelet-LLL	Firstorder	RootMeanSquared	0.974
	Wavelet-LLL	Firstorder	10Percentile	0.834
	Original	Firstorder	Median	0.994
	Wavelet-LHL	GLDM	SmallDependenceHighGrayLevelEmphasis	0.984
	Original	Firstorder	Mean	0.979
	Wavelet-LLL	Firstorder	90Percentile	0.995
	Wavelet-LLH	GLCM	ClusterShade	0.934

V-Rs	Wavelet-LLH	Firstorder	Mean	0.967
	Original	Firstorder	Mean	0.952
	Wavelet-HLL	GLSZM	SmallAreaHighGrayLevelEmphasis	0.853
	Wavelet-LLH	Firstorder	Variance	0.972
	Wavelet-HLL	GLDM	SmallDependenceHighGrayLevelEmphasis	0.946
	LoG (σ=1mm)	GLDM	DependenceNonUniformityNormalized	0.756
	Original	Firstorder	Median	0.980
	Wavelet-LLL	Firstorder	RootMeanSquared	0.936
	Wavelet-LLH	Firstorder	RobustMeanAbsoluteDeviation	0.916
	Wavelet-LLH	GLDM	DependenceVariance	0.794
	Wavelet-LLH	Firstorder	MeanAbsoluteDeviation	0.935
	Original	Firstorder	RootMeanSquared	0.956
	LoG (σ=2mm)	GLRLM	RunLengthNonUniformity	0.972
	Original	Shape	Sphericity	0.782
	Wavelet-HLH	Firstorder	MeanAbsoluteDeviation	0.988

	Wavelet-LLL	Firstorder	10Percentile	0.833
	Wavelet-HLH	Firstorder	Variance	0.875
	Wavelet-LLH	Firstorder	90Percentile	0.973
D-Rs	Wavelet-HHH	Firstorder	90Percentile	0.983
	Wavelet-HLH	Firstorder	MeanAbsoluteDeviation	0.806
	Wavelet-HHH	Firstorder	RobustMeanAbsoluteDeviation	0.956
	Wavelet-HHH	Firstorder	MeanAbsoluteDeviation	0.971
	Wavelet-HHH	Firstorder	Variance	0.993
	Wavelet-HLH	Firstorder	Variance	0.807
	Wavelet-LLH	Firstorder	Variance	0.973

LoG, Laplacian of Gaussian; GLCM, Grey-Level Co-occurrence Matrix; GLDM, Gray-level dependence matrix; GLSZM, Gray-level size zone matrix; GLRLM, Gray-level run length matrix; ICC, Interclass Correlation Coefficient.

CLAIM: Checklist for Artificial Intelligence in Medical Imaging

Section / Topic	No.	Item	☐(N/A)
**TITLE / ABSTRACT**			
	**1**	Identification as a study of AI methodology, specifying the category of technology used (e.g., deep learning)	☐
	**2**	Structured summary of study design, methods, results, and conclusions	☐
**INTRODUCTION**			
	**3**	Scientific and clinical background, including the intended use and clinical role of the AI approach	☐
	**4**	Study objectives and hypotheses	☐
**METHODS**			
Study Design	**5**	Prospective or retrospective study	☐
	**6**	Study goal, such as model creation, exploratory study, feasibility study, non-inferiority trial	☐
Data	**7**	Data sources	☐
	**8**	Eligibility criteria: how, where, and when potentially eligible participants or studies were identified (e.g., symptoms, results from previous tests, inclusion in registry, patient-care setting, location, dates)	☐
	**9**	Data pre-processing steps	☐
	**10**	Selection of data subsets, if applicable	☐
	**11**	Definitions of data elements, with references to Common Data Elements	☐
	**12**	De-identification methods	☐
	**13**	How missing data were handled	☐
Ground Truth	**14**	Definition of ground truth reference standard, in sufficient detail to allow replication	☐
	**15**	Rationale for choosing the reference standard (if alternatives exist)	☐
	**16**	Source of ground-truth annotations; qualifications and preparation of annotators	☐
	**17**	Annotation tools	☐
	**18**	Measurement of inter- and intrarater variability; methods to mitigate variability and/or resolve discrepancies	☐
Data Partitions	**19**	Intended sample size and how it was determined	☐

Insights Imaging (2024) Sun K, Wang Y, Shi Rc, Wu Sy, Wang X.

	**20**	How data were assigned to partitions; specify proportions	☐
	**21**	Level at which partitions are disjoint (e.g., image, study, patient, institution)	☐
Model	**22**	Detailed description of model, including inputs, outputs, all intermediate layers and connections	☐
	**23**	Software libraries, frameworks, and packages	☐
	**24**	Initialization of model parameters (e.g., randomization, transfer learning)	☐
Training	**25**	Details of training approach, including data augmentation, hyperparameters, number of models trained	☐
	**26**	Method of selecting the final model	☐
	**27**	Ensembling techniques, if applicable	☐
Evaluation	**28**	Metrics of model performance	☐
	**29**	Statistical measures of significance and uncertainty (e.g., confidence intervals)	☐
	**30**	Robustness or sensitivity analysis	☐
	**31**	Methods for explainability or interpretability (e.g., saliency maps), and how they were validated	☐
	**32**	Validation or testing on external data	☐
**RESULTS**			
Data	**33**	Flow of participants or cases, using a diagram to indicate inclusion and exclusion	☐
	**34**	Demographic and clinical characteristics of cases in each partition	☐
Model performance	**35**	Performance metrics for optimal model(s) on all data partitions	☐
	**36**	Estimates of diagnostic accuracy and their precision (such as 95% confidence intervals)	☐
	**37**	Failure analysis of incorrectly classified cases	**N/A**
**DISCUSSION**			
	**38**	Study limitations, including potential bias, statistical uncertainty, and generalizability	☐
	**39**	Implications for practice, including the intended use and/or clinical role	☐
**OTHER INFORMATION**			
	**40**	Registration number and name of registry	**N/A**
	**41**	Where the full study protocol can be accessed	☐
	**42**	Sources of funding and other support; role of funders	☐

Mongan J, Moy L, Kahn CE Jr. Checklist for Artificial Intelligence in Medical Imaging (CLAIM): a guide for authors and reviewers. Radiol Artif Intell 2020; 2(2):e200029.
https://doi.org/10.1148/ryai.2020200029
